# *pfhrp2* and *pfhrp3* Gene Deletions That Affect Malaria Rapid Diagnostic Tests for *Plasmodium falciparum*: Analysis of Archived Blood Samples From 3 African Countries

**DOI:** 10.1093/infdis/jiz335

**Published:** 2019-06-28

**Authors:** Rebecca Thomson, Khalid B Beshir, Jane Cunningham, Frank Baiden, Jameel Bharmal, Katia J Bruxvoort, Catherine Maiteki-Sebuguzi, Seth Owusu-Agyei, Sarah G Staedke, Heidi Hopkins

**Affiliations:** 1 London School of Hygiene and Tropical Medicine, London, United Kingdom; 2 World Health Organization, Geneva, Switzerland; 3 Department of Research and Evaluation, Kaiser Permanente Southern California, Pasadena; 4 Infectious Disease Research Collaboration, Kampala, Uganda; 5 University or Health and Allied Sciences, Kintampo Health Research Centre, Ghana

**Keywords:** *pfhrp2*, *pfhrp3*, histidine, malaria, rapid diagnostic test, deletion, mutation, Ghana, Tanzania, Uganda

## Abstract

**Background:**

Malaria rapid diagnostic tests (mRDTs) that target histidine-rich protein 2 (HRP2) are important tools for *Plasmodium falciparum* diagnosis. Parasites with *pfhrp2/3* gene deletions threaten the use of these mRDTs and have been reported in Africa, Asia, and South America. We studied blood samples from 3 African countries to determine if these gene deletions were present.

**Methods:**

We analyzed 911 dried blood spots from Ghana (n = 165), Tanzania (n = 176), and Uganda (n = 570). *Plasmodium falciparum* infection was confirmed by 18S rDNA polymerase chain reaction (PCR), and *pfhrp2/3* genes were genotyped. True *pfhrp2/3* gene deletions were confirmed if samples were (1) microscopy positive; (2) 18S rDNA PCR positive; (3) positive for merozoite surface protein genes by PCR or positive by loop-mediated isothermal amplification; or (4) quantitative PCR positive with >5 parasites/µL.

**Results:**

No *pfhrp2/3* deletions were detected in samples from Ghana, but deletions were identified in Tanzania (3 *pfhrp2*; 2 *pfhrp3*) and Uganda (7 *pfhrp2*; 2 *pfhrp3*). Of the 10 samples with *pfhrp2* deletions, 9 tested negative by HRP2-based mRDT.

**Conclusions:**

The presence of *pfhrp2/3* deletions in Tanzania and Uganda, along with reports of *pfhrp2/3*-deleted parasites in neighboring countries, reinforces the need for systematic surveillance to monitor the reliability of mRDTs in malaria-endemic countries.

## Teaser

*pfhrp2* gene deletions render *Plasmodium falciparum* parasites undetectable to malaria rapid diagnostic tests detecting histidine-rich protein 2. *pfhrp2* deletions were detected in archived blood samples from Tanzania and Uganda, while no samples from Ghana were found to be *pfhpr2*-negative.

Prompt and accurate diagnosis of malaria is crucial for malaria case-management and control and elimination programs. While malaria diagnosis was historically based on symptoms alone, since 2010 the World Health Organization (WHO) guidelines state that parasite-based diagnosis of malaria should be confirmed before treatment is given [1]. Although quality-assured microscopy remains the gold standard for diagnosis of symptomatic malaria, malaria rapid diagnostic tests (mRDTs), detecting malaria antigen(s), require less training and no specialized equipment and play an important role in malaria case management. The use of mRDTs has grown substantially since they were first developed in the 1990s, and mRDTs are currently used in the public healthcare sector in all 91 countries with malaria transmission [2].

The majority of mRDTs currently on the market detect histidine-rich protein 2 (HRP2), a parasite antigen produced throughout the life cycle of *P. falciparum*, in a blood sample [3]. In general, HRP2-based mRDTs are more sensitive and stable than mRDTs based on other *Plasmodium* antigens, and so are the mRDTs of choice in most endemic countries where *P. falciparum* malaria predominates [4].

The accuracy of HRP2-based mRDTs can be affected by factors including low parasite density (which can cause false-negative results) and antigen persisting in the bloodstream after successful treatment of a prior clinical episode (which can cause clinically false-positive results). While false-negative mRDT results have been attributed primarily to the tests’ limit of detection, recent reports have confirmed that genetic variation of *P. falciparum* can also affect mRDT performance [5, 6].

Over the past decade, *P. falciparum* strains that do not express HRP2 have been documented. The first confirmed parasites that lacked the *pfhrp2* gene were identified in the Amazon Basin in Peru in 2010, with 40% of *P. falciparum* samples testing negative for the gene [7]. Since then, similar parasites have been reported from other areas in South America [8, 9], Central America [10], India and Southeast Asia [11, 12], West Africa [13–15], and East and Central Africa [5, 16–19]. In Africa, the highest reported prevalence of *pfhrp2* deletions was in Eritrea, where 62% of samples that tested positive by microscopy were found to lack the *pfhrp2* gene [5]. While fewer studies have confirmed *pfhrp2* deletions among West African countries, a 2015 study in Ghana showed that 29% of samples lacked the *pfhrp2* gene [15]. To date, there are no published reports of *pfhrp2* deletions in Tanzania; however, *pfhrp2* deletions were reported in 6.4% of samples from children in the Democratic Republic of Congo (DRC) [16] and in 1% of microscopy-positive samples from a study in Rwanda [18]. An unpublished study from Uganda reported 1.7% *pfhrp2* deletions among 1493 microscopy-positive *P. falciparum* samples [20]. Marked heterogeneity in the prevalence of *pfhrp2* deletions within and between countries has also been described; the prevalence of *pfhrp2* deletions was reported to range from 0 to 25% between 8 states in India [11] and from 0% to 22% in different regions of DRC [16].

Parasites that do not express the HRP2 protein can cause false-negative results by HRP2-based mRDTs [6]. The HRP2 protein has an epitope that shows cross-reactivity with HRP3, also expressed by *P. falciparum*. Therefore, HRP2-based mRDTs sometimes detect infections in *pfhrp2*-deleted parasites due to the presence of HRP3, especially at higher parasite densities [21]. However, the absence of both HRP2 and HRP3 renders the parasites undetectable by HRP2-based mRDTs.

As the epidemiology of *pfhrp2* and *pfhrp3* deletions is still largely unknown, sampling strategies and molecular assessment are needed to determine the extent of these deletions in endemic areas and to assess their effect, if any, on routine clinical care of malaria patients. While awaiting the implementation of prospective surveillance, this article reports on stored *P. falciparum* samples from 3 countries: Ghana, Tanzania, and Uganda.

## METHODS

This study analyzed *P. falciparum* parasites identified in human blood samples from 3 malaria studies in Ghana, Tanzania, and Uganda. For each source study, this analysis included all available samples recorded as negative by HRP2-based mRDT and positive by microscopy, plus a random selection of available samples recorded as positive by both mRDT and microscopy, those negative by both microscopy and mRDT, and those positive by mRDT and negative by microscopy. In total, 911 samples were analyzed.

### Sample Collection

**Figure 1.**
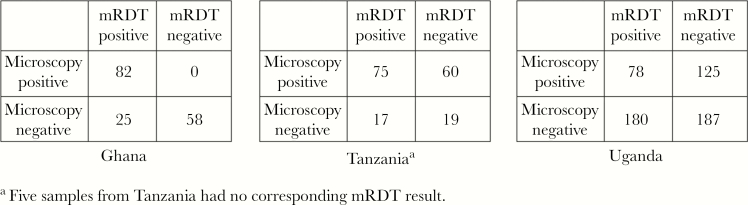
Two-by-two tables showing results of malaria rapid diagnostic tests (mRDTs) based on detection of histidine-rich protein 2 and expert microscopy for human blood samples analyzed for *pfhrp2/3* genes. ^a^Five samples from Tanzania had no corresponding mRDT result.

Samples in Ghana were collected as part of an mRDT clinical evaluation in 2009 and 2010 [22]. Information about the survey is shown in Table 1. Three hundred ninety-seven samples were collected (Table 2), of which 165 were selected for this study (Figure 1).

**Table 1.** Characteristics of Primary Studies From Which Dried Blood Spot Samples Were Selected for Analysis of *pfhrp2* and *pfhrp3*

Country	Type of Survey	Date of Sample Collection	Study Sites	Clinical Status of Participants	Age Range of Participants	mRDT Manufacturer	Estimated Entomological Inoculation Rate	Reference of Study From Which Samples Were Collected
Ghana	Health facility	2009–2010	Kintampo	Symptomatic	6–30 mo	CareStart (Access Bio)	269 ^a^	Baiden et al [22]
Tanzania	Household, health facility	2010	Mbeya, Mtwara, and Mwanza regions	Asymptomatic and symptomatic	≥6 mo	ICT Diagnostics	10.4–148.6^b^	Thomson et al [23], Bruxvoort et al [24]
Uganda	Cross-sectional	2014–2015	Jinja district	Symptomatic^c^	All ages	CareStart (Access Bio)	56.3–61.5	Staedke et al [25]

Abbreviation: mRDT, malaria rapid diagnostic test.

^a^The entomological inoculation rate was not assessed in the Ghana study. Data are from Owusu-Agyei S, Asante KP, Adjuik M, et al. Epidemiology of malaria in the forest-savanna transitional zone of Ghana. Malar J 2009; 8:220.

^b^The entomological inoculation rate was not assessed in the Tanzanian study. A range of values are presented as the study in Tanzania was conducted in 3 different regions with varying malaria transmission. Data are from Maxwell CA, Chambo W, Mwaimu M, et al. Variation of malaria transmission and morbidity with altitude in Tanzania and with introduction of alphacypermethrin treated nets. Malar J 2003; 2:28.

^c^The survey in Uganda was conducted on symptomatic and asymptomatic people, but mRDTs were performed only on symptomatic participants; therefore, samples for this study were from symptomatic people.

**Table 2.** Study Populations From Which Dried Blood Spot Samples Were Selected for Analysis of *pfhrp2* and *pfhrp3*

Study Site	mRDT Negative, Microscopy Negative	mRDT Negative, Microscopy Positive	mRDT Positive, Microscopy Negative	mRDT Positive, Microscopy Positive
Ghana	148	0	58	191
Tanzania	8319	102	1663	451
Uganda	2508	122	1395	1235

Data are presented as number.

Abbreviation: mRDT, malaria rapid diagnostic test.

Samples in Tanzania were collected during surveys in 2010 as part of an evaluation of mRDT implementation in public health facilities (IMPACT2). Samples were selected from a household survey [23] and a health facility survey [24]. In total, 10 535 samples had mRDT and microscopy results as well as dried blood spots (DBSs): 8812 from the household survey and 1723 from the health facility survey (Table 2). A total of 176 samples were selected for analysis (Figure 1).

Samples from Uganda were collected as part of the School-Based Treatment With ACT to Reduce Transmission (START-IPT) study from 2014 to 2015, a cluster-randomized trial to measure the effects of intermittent preventive treatment for malaria [25]. A total of 8922 microscopy and DBS samples were collected from cross-sectional surveys of community residents in control and intervention groups (Table 1). mRDTs were performed on participants who were febrile or had history of fever in the previous 48 hours. Unique to the Uganda study, loop-mediated isothermal amplification (LAMP) for *P. falciparum* was performed on specified proportions of samples, after DNA extraction from DBSs by standard methods with Chelex 100 Resin (Bio-Rad), resulting in 5258 samples with LAMP results. Of the samples with microscopy, LAMP, and mRDT results, 570 were selected for this study (Figure 1).

Written informed consent was obtained from all participants or participants’ caregivers. The study in Ghana was approved by the Institutional Ethics Committee of the Kintampo Health Research Centre and the ethics review committees of the Ghana Health Service. The IMPACT2 study in Tanzania was approved by the Institutional Review Board of Ifakara Health Institute. Ethical approval in Uganda was obtained from the Uganda National Council for Science and Technology; Makerere University School of Medicine Research and Ethics Committee; the School of Biological and Biomedical Sciences Ethics Committee, Durham University (United Kingdom); and the University of California, San Francisco Committee on Human Research. All 3 studies obtained ethics approval from the London School of Hygiene and Tropical Medicine (LSHTM).

### Microscopy

Thick blood smears were stained with 2% or 10% Giemsa and read in duplicate by 2 microscopists who were blinded to the initial reading and to the mRDT results. Discrepant results were resolved by a third microscopist. Parasites were counted against 200 white blood cells and were considered negative if no asexual parasites or gametocytes were found after examining 100 fields. Microscopy was performed at Kintampo Health Research Centre clinical laboratory in Ghana; Ifakara Health Institute, Bagamoyo, Tanzania; and Makerere University Molecular Research Laboratory, Mulago Hospital, Kampala, Uganda.

### Sample Storage

Samples in all 3 countries were stored in sealed plastic bags with desiccant at ambient temperature. Samples were selected in the countries of origin, and DBS samples from all countries were couriered to LSHTM in 2016. Molecular analysis was conducted at LSHTM between October 2016 and November 2017.

### Molecular Analysis

#### DNA Extraction

DNA was extracted from all DBSs using QIAsymphony according to the manufacturer’s protocol (Qiagen), using a previously published protocol [21]. A 3-mm diameter punch was taken from each DBS and placed in a deep-well plate. Buffer ATL (180 μL) and proteinase (20 μL) were added to each well and mixed at 900 rpm at 56°C for 15 minutes in a ThermoMixer. The plates were then placed into the QIAsymphony compartments for DNA extraction and the eluted DNA was stored at –20°C.

#### *Amplification of* pfhrp2 *and* pfhrp3

Parasite presence was confirmed using standard polymerase chain reaction (PCR) targeting the 18S ribosomal RNA gene of *P. falciparum* (18S rDNA) as previously published [26]. The limit of detection was 0.1 parasites/µL. For samples found positive, genotyping of *pfhrp2* and *prhrp3* (GenBank accession numbers PF3D7_0831800 and PF3D7_1372200, respectively) was then conducted using amended PCR conditions and primers published by Baker et al [27]. In brief, a seminested amplification was performed using the following conditions: 94°C for 10 minutes, then 94°C for 50 seconds, 50°C for 50 seconds, and 60°C for 1 minute. The reaction mixture contained 5 µL of extracted genomic DNA, 200 nM of each primer, 2 mM of magnesium chloride, 200 nM of each dNTP, 1X NH4 reaction buffer (Bioline), and 1.25 U of AmpliTaq Gold (ThermoFisher Scientific).

#### *Confirmation of* pfhrp2 *and* pfhrp3 *Deletion*

To confirm the deletion of *pfhrp2* and *pfhrp3* genes, PCR of 2 other single-copy genes was performed. For samples from Ghana and Tanzania, PCR of the *merozoite surface protein 1* and 2 genes (*msp1* and *msp2*, respectively) was conducted on samples that were *pfhrp2*-negative using previously published methods [28, 29]. Samples from Uganda had been tested by LAMP [25]; therefore, *msp* confirmation was not performed.

#### Quantification of Parasitemia by Quantitative PCR

The parasitemia of *pfhrp2*-negative samples was quantified by PgMET quantitative PCR (qPCR) as described in Beshir et al in 2010 [30]. The limit of detection for *pfhrp2* by this method is 5 parasites/µL [17].

#### *Classification of* pfhrp2/pfhrp3 *Genes*

Samples were considered to be truly negative for *pfhrp2* or *pfhrp3* if deletions were identified as above and (1) they were positive by microscopy, and (2) they tested positive by 18S rDNA PCR, and (3) *msp* genes were detected by PCR (Ghana, Tanzania) or the sample was positive by LAMP (Uganda). Furthermore, only samples above the limit of detection of 5 parasites/µL by qPCR were considered true *pfhrp2/3*-negatives, as samples below this parasite density may have given false-negative results by *pfhrp2/3* PCR.

## RESULTS

### Percentage of Samples Testing Positive for *P. falciparum* in Study Samples, by Detection Method

**Figure 2.**
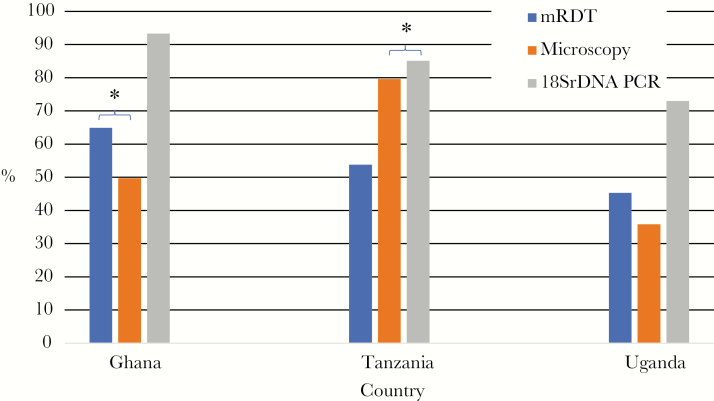
Percentage of samples positive for *Plasmodium falciparum* in study samples, by detection method. *Denotes κ value of ≥0.6, indicating good agreement between diagnostic methods. Abbreviations: mRDT, malaria rapid diagnostic test; PCR, polymerase chain reaction.

Among the samples from Ghana, 107 of 165 (64.9%) were recorded as positive by mRDT and 82 of 165 (49.7%) by microscopy (Figure 2). In Tanzania, 72 of 171 (53.8%) samples were recorded as positive by mRDT, whereas 140 of 176 (79.6%) were positive by microscopy (5 samples did not have mRDT results). Of the 570 Ugandan samples, 258 of 570 (45.3%) were recorded as positive by mRDT, and 203 (35.6%) were positive by microscopy.

Microscopy and mRDT results for the samples included in this analysis are presented in Figure 1. Among the samples available from Ghana, none were recorded as positive by microscopy and negative by mRDT. In Tanzania, about one-third of the samples selected for analysis were positive by microscopy and negative by mRDT (60/171 [35.1%]), whereas in Uganda 125 of 570 (21.9%) samples were recorded as positive by microscopy and negative by RDT.

Microscopy-determined parasite density in Ghana ranged from 371 to 1 500 000 parasites/µL (mean, 128 505; median, 37 960.5). In Tanzania, the range was 2–9249 parasites/µL (mean, 1079; median, 60.5). Microscopy-determined parasite densities were not recorded for Ugandan samples.

### Presence of *pfhrp2/pfhrp3* Gene Deletions

**Figure 3.**
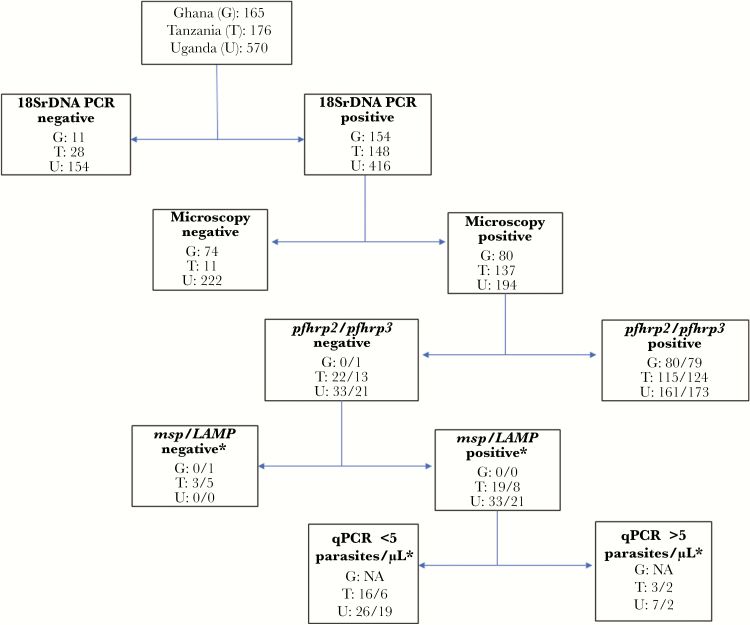
Flow diagram showing process of determining *pfhrp2* and *pfhrp3* gene deletions in blood samples from studies in 3 African countries. *The first number in each row denotes the number of samples among *pfhrp2*-negative samples; the second number denotes the number among *pfhrp3*-negative samples. Abbreviations: G, Ghana; LAMP, loop-mediated isothermal amplification; *msp*, merozoite surface protein; PCR, polymerase chain reaction; qPCR, quantitative polymerase chain reaction; T, Tanzania; U, Uganda.

Of the 165 samples from Ghana, 154 (93.3%) tested positive by 18S rDNA and 80 (48.5%) tested positive by both 18S rDNA and microscopy (Figure 3). All 80 samples tested positive for *pfhrp2*, and only 1 sample tested negative for *pfhrp3*. No Ghanaian sample was both positive by microscopy and negative by mRDT (Table 3).

**Table 3.** Samples With *pfhrp2* and *pfhrp3* Deletions Among Polymerase Chain Reactive–Positive *Plasmodium falciparum* Samples, by Country of Origin and Results of Microscopy and Histidine-Rich Protein 2–Based Malaria Rapid Diagnostic Test

Samples	mRDT Negative, Microscopy Negative	mRDT Negative, Microscopy Positive	mRDT Positive, Microscopy Negative	mRDT Positive, Microscopy Positive
No. of PCR-positive samples among all samples analyzed				
Ghana	50	0	24	80
Tanzania	7	57	4	75
Uganda	176	116	46	78
*pfhrp* gene deletion status among PCR-positive samples				
Ghana				
No deletion	50	0	24	80
*pfhrp2*^–^/*pfhrp3*^*+*^	0	0	0	0
*pfhrp2*^*+*^/*pfhrp3*^*–*^	0	0	0	0
*pfhrp2*^*–*^/*pfhrp3*^*–*^	0	0	0	0
Tanzania				
No deletion	7	55	4	74
*pfhrp2*^*–*^/*pfhrp3*^*+*^	0	1	0	0
*pfhrp2*^*+*^/*pfhrp3*^*–*^	0	0	0	0
*pfhrp2*^*–*^/*pfhrp3*^*–*^	0	1	0	1
Uganda				
No deletion	176	109	46	78
*pfhrp2*^*–*^/*pfhrp3*^*+*^	0	5	0	0
*pfhrp2*^*+*^/*pfhrp3*^*–*^	0	0	0	0
*pfhrp2*^*–*^/*pfhrp3*^*–*^	0	2	0	0

Abbreviations: mRDT, malaria rapid diagnostic test; PCR, polymerase chain reaction.

Of the 176 samples from Tanzania, 148 (85.1%) were positive by 18S rDNA PCR, of which 137 were positive by microscopy. After applying the confirmation criteria for *pfhrp2/3* deletions, 3 samples were found to have *pfhrp2* deletions. Two samples had *pfhrp3* deletions; both of these also had *pfhrp2* deletions (Table 3).

Of the 570 samples from Uganda, 416 (73.0%) were positive by PCR, of which 194 were microscopy positive. After applying the confirmation criteria, 7 samples were found to have *pfhrp2* deletions. Two samples had *pfhrp3* deletions; both of these were also negative for *pfhrp2*.

Overall, 9 of the 10 *pfhrp2*-negative samples tested positive by microscopy and negative by mRDT (Table 3). Six of these samples had an intact *pfhrp3* gene while 4 did not. One sample from Tanzania was positive by both the ICT Diagnostics mRDT and microscopy, and was negative for *pfhrp3*. The parasite concentration of these 10 samples ranged from 7.3 to 3800 parasites/µL by qPCR. No sample was negative for *pfhrp3* and positive for *pfhrp2*.

## DISCUSSION

*Plasmodium falciparum* parasites lacking the genes coding for histidine-rich proteins, which are detected by commonly used mRDTs, pose a threat to malaria control and elimination programs. This report presents an analysis of *pfhrp2/3* in archived human blood samples from 3 African countries, alongside microscopy and mRDT results obtained in the primary studies from which the samples were drawn. Molecular analysis identified low levels of *pfhrp2* and *pfhrp3* gene deletions in samples from Tanzania (collected in 2010) and Uganda (2014–2015), whereas no evidence of deletions was found in samples from Ghana (2009–2010).

Of the 10 *pfhrp2*-negative samples identified in this study, 9 were recorded as negative by HRP2-based mRDT, 7 by the mRDT used in Uganda, and 2 by the mRDT in Tanzania. The exception was 1 sample from Tanzania, which was negative for both *pfhrp2* and *pfhrp3*, but positive by HRP2-based mRDT; possible explanations for this could be a data recording error, or a false-positive mRDT due to cross-reactions with human antimouse antibodies or rheumatoid factor [31]. All mRDTs used in the original studies performed well in the WHO product testing rounds of the corresponding study years; however, the panel detection score of the mRDTs used in Uganda and Ghana was higher than that of the mRDT used in Tanzania [4, 32], which might explain, at least partly, why parasite prevalence by mRDT was lower than by microscopy in Tanzania. Also, most samples from Tanzania were from asymptomatic people, whereas samples from the other 2 countries were from symptomatic patients, resulting in lower parasite density among Tanzanian *P. falciparum*–positive samples.

Of the 9 *pfhrp2*-negative samples that tested negative by mRDT, 6 had intact *pfhrp3*. While it is well-documented that HRP2-based mRDTs may give false-negative results in the absence of *pfhrp2* [5, 11, 13], it has also been found that cross-reaction with epitopes on HRP3 can produce positive mRDT results [7, 15, 27], especially at concentrations >1000 parasites/µL [17]. Cross-reactivity of HRP3 on HRP2-based RDTs has also been shown to vary between mRDT brands [33]. In this study, the parasite densities in the 6 *pfhrp2*-negative/*pfhrp3*-positive samples ranged from 7.3 to 69.3 parasites/µL, likely too low to be detected by mRDTs even if HRP3 was present. Parasites with deletions in both *pfhrp2* and *pfhrp3* genes are undetectable by HRP2-based RDTs [34], and therefore the presence of *pfhrp3* deletions in these populations is significant.

In this study a true *pfhrp2*-negative sample was defined as the absence of *pfhrp2* in a sample that tested positive for malaria by microscopy and positive for *P. falciparum* either by LAMP or 2 other single-copy genes. This produces a conservative estimate of *pfhrp2* deletion; some other studies have reported *pfhrp2* deletions based only on failure to amplify the *pfhrp2* gene by PCR, without also confirming parasite presence with 2 other single-copy genes by PCR, which may produce more alarming results [8, 9, 35]. We also chose a qPCR cutoff of 5 parasites/µL, the limit of detection for *pfhrp2* PCR, to determine true *pfhrp2*-negative samples [17]. Any samples with a parasite density below this threshold may have produced false-negatives for *pfhrp2* PCR and could not be confirmed as true *pfhrp2* negatives. While the majority of published studies have not applied this criterion in their identification of *pfhrp2* deletions, doing so produces a conservative and more confident definition of *pfhrp2* deletion [34]. Indeed, the number of samples found to be *pfhrp2* negative would have been higher without this cutoff (Figure 3).

While this is the first report of *pfhrp2* gene deletions in Tanzania, findings from neighboring Rwanda [36], Kenya [21], DRC [16], and nearby Eritrea [5, 19] indicate that the phenomenon is present in the region. There are a few reports of *pfhrp2* gene deletions in other countries in West Africa, including a study using archived samples from Mali [13] and a study in Senegal [14]. Although our study did not show any deletions in Ghana, 2 other studies in Ghana have reported alarming results of 29% [15] and 75% [37], although the latter was among a small sample of only 8 children. Samples from the former study were collected in 2015, from Gold Coast and Accra, both in the south of the country, while samples from the latter study were collected in Accra, also in 2015. Samples in our study were collected in 2010 in Kintampo, in the middle of Ghana, so the differing areas and times of sample collection could explain the different findings.

 Of note, the majority of these studies were not designed specifically to investigate the epidemiology of *pfhrp2/3* deletions; deletion analysis was conducted on samples that had been collected to address other primary objectives, which is also the case for the study reported here. Although reports of *pfhrp2/3* deletions in neighboring or nearby countries are suggestive, prevalence within a geographic area can be highly heterogenous [11, 16], and the design of surveillance efforts should take this into account.

The *pfhrp2* gene amino acid sequence and repeats have been shown to vary substantially across different geographic regions [38]. This study looked only at presence vs absence of *pfhrp2*/*3* genes. Genomic sequencing of exons and flanking regions would provide more information on sequence diversity among these samples. Even *pfhrp2*-positive samples may harbor genetic diversity with implications for mRDT detection. Although diversity in the *pfhrp2* gene has not been found to affect mRDT affinity in samples with parasite densities of clinical significance [11, 39], it has been shown to affect mRDT results at densities <200 parasites/µL [27].

The phenomenon of *pfhrp2/3* gene deletions poses a substantial threat to malaria control and could reverse the gains made through the rapid expansion of mRDT uptake over the past decade [40]. Prescriber adherence to test results, especially negative test results, has been a key focus of mRDT implementation efforts to date [41, 42]. False-negative mRDT results lead to underdiagnosis of malaria, and if patients who are infected but test negative do not receive antimalarial treatment, severe disease and even death may result; the *pfhrp2*-deleted parasites in their bloodstream may then be taken up by female *Anopheles* mosquitoes and transmitted to others [6]. Models have demonstrated that newly introduced *pfhrp2*-negative parasites can spread rapidly though a community if HRP2-based mRDTs are the only diagnostic tool used to guide treatment practices [40]. Using publicly available genomic data generated from genetic crosses, the absence of fitness cost for *hrp2*-negative parasites has recently been reported [43].

In malaria-endemic countries, assessment and surveillance of *pfhrp2/3* deletions and their impact must be undertaken effectively and efficiently, alongside multiple other public health and malaria control priorities. To this end, WHO has published a protocol for implementing surveys designed to measure *pfhrp2*-deleted parasites among malaria suspects [44]. WHO guidelines state that if the prevalence of *pfhrp2* gene deletions that cause false-negative HRP2-based RDT results in a representative sample is higher than 5%, HRP2-based mRDTs should be replaced with a new diagnostic tool [44]. In such cases, mRDTs that target other antigens, such as those detecting pan-LDH or Pf-pLDH, may be considered. However, pLDH-based mRDTs are generally less sensitive and heat stable than HRP2-based RDTs, and this trade-off must be weighed in considering a switch. The 5% threshold in the WHO guidance is estimated to be the prevalence at which the benefits of non-HRP2-based diagnostics for detecting *pfhrp2*-deleted parasites outweighs the reduced sensitivity of these tools to detect wild-type parasites.

This study has several limitations. The blood samples analyzed were collected as part of other malaria studies that were not designed to study *pfhrp2/3* deletions nor to measure prevalence of these mutations. DBS samples were purposively selected from the available samples and were not representative of the total original study populations. Furthermore, the samples were taken from different human populations, including a household survey of asymptomatic individuals and exit interviews of febrile patients who sought care at healthcare facilities; in Ghana these surveys targeted children, whereas in Tanzania and Uganda they targeted individuals of all ages. Samples were collected at different time points, from 2010 in Ghana to 2015 in Uganda, which may affect the findings if the epidemiology of gene deletions has changed over time. This makes it impossible to directly compare results across the 3 countries. Samples had been stored for several years before molecular analysis in nonrefrigerated conditions; however, a set of criteria was followed to determine *pfhrp2/3* deletions to compensate for this. This molecular analysis focused on exon 2, as this is the main part of the gene that affects RDT performance. However also targeting the region across exon 1 and flanking genes would provide greater confirmatory evidence of gene deletions and enable detection of partial gene deletions on chromosome breaking points. Rather than measuring prevalence of gene deletions, this study serves as one indicator, using rigorous laboratory methods to determine whether any mutated parasites are present in available samples from the study areas.

This report documents the presence of *pfhrp2/3* gene deletions in *P. falciparum* in archived blood samples from 2 East African countries, Tanzania and Uganda. Further studies and surveillance will be essential to better understand the epidemiology of these parasites, as well as to guide future decisions about diagnostic tools and strategies. Although no conclusions about the prevalence of *pfhrp2/3* deletions can be drawn from this study, the fact that only a few deleted parasites were identified suggests that HRP2-based mRDTs are still a valid diagnostic tool in these countries. However, together with other reports documenting the presence and potential spread of such parasites in nearby areas, this study reinforces the WHO call for systematic surveillance to monitor the reliability of mRDTs [44].

## Notes

***Acknowledgments.*** We gratefully acknowledge the assistance of the clinical staff and patients in all of the studies contributing data for this analysis. We thank Daniel Chandramohan, Catherine Goodman, Patrick Kachur, David Schellenberg, and Jayne Webster for leading the primary studies from which the samples analyzed in this report were taken; Levi Mugenyi, Sam Nsobya, Emmanuel Ruhamya, Andrea Rehman, Matilda Tivura, and David Dosoo for assistance in selecting and shipping the samples; Paul Snell and Sham Lal for development and management of the central data repository; and Colin Sutherland for advice and comments on the submitted manuscript.

***Disclaimer.*** The study funders had no role in the study design, implementation, analysis, manuscript preparation, or decision to submit this article for publication.

***Financial support.*** The Ghana and Tanzania primary studies and this analysis were supported by the ACT Consortium, which was funded through a grant from the Bill & Melinda Gates Foundation to the London School of Hygiene and Tropical Medicine. START-IPT was funded by the Medical Research Council, the Department for International Development, and the Wellcome Trust under the Joint Global Health Trials scheme (MR/K00736X/1). This award is part of the EDCTP2 program supported by the European Union. Part of the laboratory work was supported by the Rosemary Weir prize awarded to K. B. B. by the Department of Infection and Biology, London School of Hygiene and Tropical Medicine.

***Potential conflicts of interest.*** All authors: No reported conflicts of interest.
